# Sepsis patients with complication of hypoglycemia and hypoalbuminemia are an early and easy identification of high mortality risk

**DOI:** 10.1007/s11739-019-02034-2

**Published:** 2019-02-07

**Authors:** Makoto Furukawa, Kosaku Kinoshita, Junko Yamaguchi, Satoshi Hori, Atsushi Sakurai

**Affiliations:** Division of Emergency and Critical Care Medicine, Department of Acute Medicine, Nihon University School of Medicine, 30-1, Oyaguchi Kami-cho, Itabashi-ku, Tokyo, 173-8610 Japan

**Keywords:** Sepsis, Hypoglycemia, Hypoalbuminemia, Mortality, Outcome prediction

## Abstract

Either hypoglycemia or hypoalbuminemia alone is an independent condition associated with increased risk of mortality in critical illness. This study evaluates whether the mortality risk increases in septic patients if these conditions are combined. Patients admitted to our hospital from 2008 to 2015 who satisfied the definition of sepsis were targeted (*n* = 336). We classified cases into three groups based on blood glucose (BG) level measured at admission: hypoglycemia (Hypo-G; BG < 80 mg/dl), intermediate glycemia (Inter-G; 80–199 mg/dl), and hyperglycemia (Hyper-G; ≥ 200 mg/dl) group, and then estimated mortality. We also compared the clinical data of these glycemic groups in combination with hypoalbuminemia (Hypo-A) or Inter-G with non-hypoalbuminemia (Inter-G + Nonhypo-A), as a secondary analysis. Diagnostic cut-off level of Hypo-A (< 2.8 mg/dl) was determined using the ROC curve between blood albumin and mortality. In Hypo-G group (*n* = 40), APACHE II/SOFA scores are significantly higher than in the Inter-G (*n* = 196) and Hyper-G groups (*n* = 100). Mortality is 52.5% in the Hypo-G and 60.0% in the Hypo-G with Hypo-A (Hypo-G + Hypo-A) groups. Significantly higher APACHE II or SOFA scores and mortality are observed in the Hypo-G + Hypo-A group compared to the Inter-G + Nonhypo-A group. A higher mortality risk is observed in cases with Hypo-G + Hypo-A (OR 5.065) than those with Hypo-G (OR 3.503), Inter-G (OR 1.175), Hyper-G (OR 1.756) or Hypo-A (OR 3.243), calculated by a single logistic-regression analysis. Hypo-G + Hypo-A in patients with sepsis is related to higher ICU mortality. Physicians should be keenly aware of these conditions to provide immediate intensive treatment after admission of septic patients.

## Background

Hypoglycemia during hospitalization is associated with an increased hospital stay and contributes to a poorer outcome in diabetes patients in general medical wards in diabetic patients [1, 2]. While clinical evidence indicates that hypoglycemia following infectious illness in non-diabetic patients, such as pneumonia, is linked to poor outcomes [3, 4], hypoalbuminemia is also often observed in critically ill patients, and is related to outcome [5]. Recently, we report that low albumin is a significant predictor related to poor outcome in septic patients [6]. These studies have addressed either hypoglycemia or hypoalbuminemia alone as an independent risk factor that affects mortality in patients with critical illness. The significance of hypoglycemia with hypoalbuminemia in septic patients, however, has yet to be clarified. Early identification of high mortality risk in septic patients is an important clinical issue to improve overall outcome [7–9]. We hypothesize that hypoglycemia with hypoalbuminemic conditions may be related to an increased risk of mortality in septic patients. The objective of this study was to determine whether hypoglycemia with hypoalbuminemia at the time of admission might predict a higher risk of mortality in patients with sepsis.

## Methods

This study was approved by the Clinical Research Review Committee of Nihon University School of Medicine (RK-160808-1) and was designed as a single-institution retrospective observational investigation using the database of patients treated for sepsis at our hospital. Sepsis is defined as infection plus systemic manifestations of infection [10], in patients admitted to the intensive care unit (ICU) of this hospital between January 2008 and December 2015.

In this study, data on the patients were obtained from the database system at our hospital. Every morning, team members (physicians, nurses, a pharmacologist, and an emergency medical technician) in the emergency department held a conference to review and discuss the details of the clinical record of each patient newly transferred to our department, including many factors related to outcome. All data for this study were obtained from the database and our patients’ clinical records.

Cases in which treatment for sepsis had already began in another hospital before admission to our hospital, cases of transfer after only resuscitation treatment at our hospital, and cases where details regarding pre-hospital events were incomplete, were excluded from this study.

To confirm infectious illness diagnosis, blood culture and whole body CT scans of the infection source were performed in this study.

Peripheral whole blood was collected from patients at admission. Patient information and laboratory data were recorded, including age, gender, Acute Physiology and Chronic Health Evaluation (APACHE) II score [11], and sequential organ failure assessment (SOFA) sore [12].

Cases were classified into three groups based on blood glucose level measured at admission: a hypoglycemia (hypo-G) group (blood glucose level < 80 mg/dl), a hyperglycemia (hyper-G) group (blood glucose level ≥ 200 mg/dl), and an intermediate glycemia (inter-G) group (blood glucose level 80–199 mg/dl) [13, 14].

Blood albumin levels were used to determine hypoalbuminemia associated with mortality according to receiver operating characteristic (ROC) curve-derived cut-off values. According to the ROC curve, the area under the curve (AUC) was 0.68 for the blood albumin (sensitivity 0.72, 1-specificity 0.43) and the value of blood albumin concentration was 2.8 mg/dl. In this study, a blood albumin level < 2.8 mg/dl is defined as hypoalbuminemia (Hypo-A).

As a secondary analysis, the effect of hypoalbuminemia in different combinations of hyperglycemia or hypoglycemia at the time of admission was estimated in septic patients. Glucose levels in patients with hypoalbuminemia at the time of admission were divided into separate groups and analyzed. Group distribution is as follows—Hypo-G + Hypo-A: Patients with a glycemic level < 80 mg/dl and blood albumin < 2.8 mg/dl, Inter-G + Hypo-A: patients with a glycemic level between 80 and 199 mg/dl and blood albumin < 2.8 mg/dl, and Hyper-G + Hypo-A: Patients with glycemic level ≥ 200 mg/dl and blood albumin < 2.8 mg/dl. As the control group, Inter-G + Nonhypo-A (non-hypoalbuminemia) was defined as patients with a glycemic level between 80 and 199 mg/dl and blood albumin over 2.8 mg/dl. In each Hypo-A, Inter-G + Nonhypo-A, Hypo-G + Hypo-A, Inter-G + Hypo-A and Hyper-G + Hypo-A group, there were some cases which were included in more than one group.

Outcome was evaluated at 28 ICU days or when the patient was discharged or transferred from our hospital.

### Statistical analysis

All analyses were conducted using SPSS (IBM Statistics Version 22, Chicago, IL, USA) and JMP ver. 11.0 (SAS Institute, Cary, NC, USA). Data were presented as mean values [standard deviation (SD)] or number of cases (%). *p* < 0.05 was set as the definition of statistical significance.

Continuous variables were compared using Student’s *t* test or the Mann–Whitney *U* test as appropriate. The Pearson exact test was performed for categorical variables. Physiological data from each glycemic condition group were compared by one-way analysis of variance (ANOVA) or Kruskal–Wallis analysis. Subsequently, Tukey–Kramer or Steel–Dwass’s post hoc test was performed.

Outcome was predicted by multiple logistic-regression and calculating the odds ratios (OR) and 95% confidence interval (CI). Variables with *p* values < 0.2 by bivariate analysis were then introduced into the multivariate model [15]. The multivariate model was used to determine previously described clinical factors related to outcome as explanatory variables.

Multicollinearity, assessed using variance inflation factors [16], was detected among age, bilirubin, platelet, creatinine and APACHE II score or SOFA score, and among blood glucose levels (each glycemic group), and between blood albumin levels (cases with hypoalbuminemia); these variables were appropriately chosen in the multivariate model.

The Hypo-A, Inter-G + Nonhypo-A, Hypo-G + Hypo-A, Inter-G + Hypo-A and Hyper-G + Hypo-A groups were not included in the multivariate model because of multicollinearity due to some patients being in multiple groups. Finally, predicted outcome of patients in each glycemic condition was considered using a single regression analysis and the relationship between each glycemic condition group and hypoalbuminemia at admission was estimated.

## Results

Five hundred and thirty consecutive cases of patients with sepsis were enrolled during the study period. After excluding 184 who had already begun the treatment at another hospital and 8 patients with incomplete data, 336 septic patients (204 males, 132 females) were included in this study.

The origins of the infection focus of the study patients were lung (pneumonitis; *n* = 176), urine tract (pyelonephritis; *n* = 53), abdomen (perforation of colon, *n* = 31), soft tissue (*n* = 19), central nervous system (*n* = 6), endocardial system (endocarditis; *n* = 2), and others, including unknown origin (*n* = 49) (Table 1).

**Table 1:** Infection focus in each glycemic group

Infection focus	Hypo-G	Inter-G	Hyper-G
Lung (*n* = 176)	18 (10.2%)	103 (58.5%)	55 (31.3%)
Urine tract (*n* = 53)	6 (11.3%)	29 (54.7%)	18 (34.0%)
Abdomen (*n* = 31)	3 (9.8%)	17 (54.8%)	11 (35.5)
Soft tissue (*n* = 19)	0 (0%)	12 (63.2%)	7 (36.8%)
Central nervous system (*n* = 6)	0 (0%)	4 (66.7%)	2 (33.3%)
Endocardial systems (*n* = 2)	0 (0%)	0 (0%)	2 (100%)
Other (*n* = 49)	12 (24.5%)	29 (59.2%)	8 (16.3%)

*Hypo-G* hypoglycemia group (blood glucose level < 80 mg/dl), *Hyper-G* hyperglycemia group (blood glucose level ≥ 200 mg/dl), *Inter-G* intermediate glycemia group (blood glucose level = 80–199 mg/dl), *Others* including cases of unknown origin

Background and outcome of this study could be demonstrated when the patients were divided into two groups: a survival group and a non-survival group (Table 2). The significant higher values in APACHE II/SOFA score, higher GOT, higher BUN, lower blood albumin level, and metabolic acidosis were observed in the non-survival group although total bilirubin and blood glucose levels between groups are not significantly different (Table 2a). When the patients are divided into three groups based on blood glucose level measured at admission, the number of each group is as follows: 40 (11.9%) patients in the hypo-G group, 196 (58.3%) patients in the inter-G group, 100 (29.8%) patients in the hyper-G group. Distribution of all glycemic groups is shown in Table 2b. A significantly higher number of non-survivors is observed in the Hypo-G, Hyper-G, Hypo-A, Hypo-G + Hypo-A and Inter-G + Hypo-A groups, excluding the inter-G, Inter-G + Nonhypo-A and Hyper-G + Hypo-A groups. Conversely, Inter-G + Nonhypo-A group shows a significantly higher number of survivors compared to non-survivors.

**Table 2:** Comparison of survivors and non-survivors

	All (*n* = 336)	Survivors (*n* = 244)	Non- survivors (*n* = 92)	*p* value
(A) Parameters				
Age	73.9 ± 14.7	72.8 ± 15.3	76.7 ± 12.9	0.0337
Gender (male; %)	204 (61%)	151 (62%)	53 (58%)	0.4742
APACHE II score	23.1 ± 7.3	21.6 ± 6.5	27.0 ± 7.8	< 0.0001
SOFA score	7.5 ± 3.1	6.9 ± 2.7	9.0 ± 3.5	< 0.0001
WBC (× 10^3^/μl)	12.4 ± 8.3	12.8 ± 8.1	11.5 ± 8.7	0.2175
Hemoglobin (g/dl)	11.9 ± 2.7	12.1 ± 2.7	11.5 ± 2.6	0.0828
Hematocrit (%)	35.6 ± 7.7	35.9 ± 7.7	34.6 ± 7.6	0.0738
Platelet (× 10^4^/μl)	21.5 ± 11.8	20.4 ± 13.8	21.9 ± 11.0	0.0347
Albumin (g/dl)	2.84 ± 0.74	2.97 ± 0.72	2.51 ± 0.67	< 0.0001
T. bilirubin (mg/dl)	1.09 ± 1.32	1.04 ± 1.30	1.24 ± 1.36	0.4334
GOT (U/l)	170.9 ± 760.7	116.8 ± 279.9	314.4 ± 1375.5	0.0013
GPT (U/l)	77.2 ± 304.3	57.9 ± 125.4	128.3 ± 543.4	0.1427
Na (mEq/l)	139.53 ± 8.77	139.55 ± 7.99	139.49 ± 10.61	0.8693
K (mEq/l)	4.39 ± 1.05	4.28 ± 1.01	4.69 ± 1.10	0.0013
BUN (mg/dl)	49.8 ± 40.0	45.9 ± 39.1	60.4 ± 40.6	< 0.0001
Creatinine (mg/dl)	2.04 ± 2.0	1.96 ± 2.08	2.25 ± 1.75	0.0843
Lactate (mmol/l)	5.38 ± 4.99	4.64 ± 4.73	7.37 ± 5.13	< 0.0001
HCO^3−^ (mmol/l)	19.6 ± 5.8	20.0 ± 5.9	18.4 ± 5.7	0.0211
CRP (mg/dl)	13.6 ± 11.5	12.6 ± 10.8	15.3 ± 12.3	0.0852
B.G (mg/dl)	186.4 ± 145.7	191.2 ± 137.4	173.9 ± 165.8	0.3741
(B) Glycemic groups				
Hypo-G	40/336 (11.9%)	19/244 (7.8%)	21/92 (22.8%)	0.0001
Inter-G	196/336 (58.3%)	145/244 (59.4%)	51/92 (55.4%)	0.5081
Hyper-G	100/336 (29.8%)	80/244 (32.8%)	20/92 (21.7%)	0.0483
Hypo-A	146/336 (43.4%)	87/244 (35.7%)	59/92 (64.1%)	< 0.0001
Inter-G + Nonhypo-A	113/336 (33.6%)	95/244 (38.9%)	18/92 (20.0%)	0.0005
Hypo-G + Hypo-A	24/336 (7.1%)	9/244 (3.7%)	15/92 (16.3%)	< 0.0001
Inter-G + Hypo-A	94/336 (28.0%)	59/244 (24.2%)	35/92 (38.0%)	0.0116
Hyper-G + Hypo-A	37/336 (11.0%)	27/244 (11.1%)	10/92 (10.9%)	0.9592

*APACHE II* Acute Physiology and Chronic Health Evaluation II, *SOFA score* Sequential Organ Failure Assessment score, *T. bilirubin* total bilirubin, *WBC* white blood cell, *CRP* C-reactive protein, *Hypo-G* hypoglycemia group (blood glucose level < 80 mg/dl), *Hyper-G* hyperglycemia group (blood glucose level ≥ 200 mg/dl), *Inter-G* intermediate glycemia group (blood glucose level = 80–199 mg/dl), *Hypo-A* hypoalbuminemia (blood albumin < 2.8 mg/dl), *Nonhypo-A* non-hypoalbuminemia (blood albumin ≥ 2.8 mg/dl or more), *Inter-G + Nonhypo-A* patients with a glycemic level between 80 and 199 mg/dl and blood albumin 2.8 mg/dl or more, *Hypo-G + Hypo-A* patients with a glycemic level < 80 mg/dl and blood albumin < 2.8 mg/dl, *Inter-G + Hypo-A* patients with a glycemic level between 80 and 199 mg/dl and blood albumin < 2.8 mg/dl, *Hyper-G + Hypo-A* patients with a glycemic level ≥ 200 mg/dl and blood albumin < 2.8 mg/dl, In each Hypo-A, Inter-G + Nonhypo-A, Hypo-G + Hypo-A, Inter-G + Hypo-A and Hyper-G + Hypo-A group, there were some cases which were included in more than one group

Independent predictors of non-survivors are shown in Table 3. Multiple logistic regression analysis of the initial laboratory data at admission shows that low albumin (OR 0.4617; 95% CI 0.2769–0.7549, *p* = 0.0019), lactate (OR 1.0703; 95% CI 1.0074–1.1416, *p* = 0.0027) or, the presence of hypoglycemia (Hypo-G; OR 3.1424; 95% CI 1.5649–6.3638, *p* = 0.0014) is associated with higher mortality. Other independent predictors of high mortality risk do not demonstrate a correlation.

**Table 3:** Independent predictors of non-survival

Predictors	Odds ratio	95% CI	*p* value
(A) Parameters			
Age	1.018	0.9959–1.0428	0.1129
Gender (male; %)	–	–	–
APACHE II score	1.0481	0.9877–1.11245	0.1207
SOFA score	1.081	0.9491–1.2362	0.2413
WBC (× 10^3^/μl)	NA	–	–
Hemoglobin (g/dl)	0.9917	0.8826–1.1147	0.8884
Hematocrit (%)	NA	–	–
Platelet (× 10^4^/μl)	NA	–	–
Albumin (g/dl)	0.4617	0.2769–0.7549	0.0019
T. bilirubin (mg/dl)	NA	–	–
GOT (U/l)	1.002	0.9993–1.0053	0.1704
GPT (U/l)	0.9964	0.9889–1.00258	0.2805
Na (mEq/l)	NA	–	–
K (mEq/l)	NA	–	–
BUN (mg/dl)	1.0027	0.9951–1.0101	0.4826
Creatinine (mg/dl)	NA	–	–
Lactate (mmol/l)	1.0703	1.0074–1.1416	0.0270
HCO^3−^ (mmol/l)	NA	–	–
CRP (mg/dl)	1.0095	0.9830–1.0365	0.4808
B.G (mg/dl)	–	–	–
(B) Glycemic groups			
Hypo-G	3.1424	1.5649–6.3638	0.0014
Inter-G	–	–	–
Hyper-G	0.7108	0.3894–1.2601	0.2462
^a^Hypo-A	NA	–	–
^a^Inter-G + Nonhypo-A	NA	–	–
^a^Hypo-G + Hypo-A	NA	–	–
^a^Inter-G + Hypo-A	NA	–	–
^a^Hyper-G + Hypo-A	NA	–	–

All variables with *p* value < 0.2 in the bivariate model (Table 1) were next considered in the multivariate model (multiple logistic-regression analysis)

SOFA and APACHE II scores were comprised of variables WBC, Hematocrit, Platelet, T. bilirubin, Na, K, Creatinine, and HCO^3−.^ Hence, multiple logistic-regression analysis was not used for the multivariate model due to multicollinearity

^a^Since some cases in each Hypo-A, Inter-G + Nonhypo-A, Hypo-G + Hypo-A, Inter-G + Hypo-A, and Hyper-G + Hypo-A group are included in more than one group, multiple logistic-regression analysis could also not be calculated. These parameters were indicated as “NA” in the table

*NA* not applicable, *APACHE II* Acute Physiology and Chronic Health Evaluation II, *SOFA score* Sequential Organ Failure Assessment score, *T. bilirubin* total bilirubin, *WBC* white blood cell, *CRP* C-reactive protein, *Hypo-G* hypoglycemia group (blood glucose level < 80 mg/dl), *Hyper-G* hyperglycemia group (blood glucose level ≥ 200 mg/dl), *Inter-G* intermediate glycemia group (blood glucose level = 80–199 mg/dl), *Hypo-A* hypoalbuminemia (blood albumin < 2.8 mg/dl), *Nonhypo-A* non-hypoalbuminemia (blood albumin ≥ 2.8 mg/dl or more), *Inter-G + Nonhypo-A* patients with a glycemic level between 80 and 199 mg/dl and blood albumin 2.8 mg/dl or more, *Hypo-G + Hypo-A* patients with a glycemic level < 80 mg/dl and blood albumin < 2.8 mg/dl, *Inter-G + Hypo-A* patients with a glycemic level between 80 and 199 mg/dl and blood albumin < 2.8 mg/dl, *Hyper-G + Hypo-A* patients with a glycemic level ≥ 200 mg/dl and blood albumin < 2.8 mg/dl

Distribution of all glycemic groups for patients with and without hypoalbuminemia is shown in Table 4. All groups are compared with the Inter-G + Nonhypo-A group (control). Some cases were included in more than one group. APACHE II scores are significantly higher in the Hypo-G, Hyper-G, Hypo-A, and Hypo-G + Hypo-A groups compared to the control group, but SOFA scores are significantly higher than those of the Hypo-G and Hypo-G + Hypo-A groups, found using a multiple comparison test. Mortality is 52.5% in the Hypo-G, 40.2% in the Hypo-A, and 60.0% in the Hypo-G + Hypo-A group. Hypo-G + Hypo-A group shows a significantly higher rate (*p* < 0.0001) than the control group (15.2% in the Inter-G + Nonhypo-A group) using the Pearson exact test.

**Table 4:** Distribution of various glycemic conditions in patients with or without hypoalbuminemia

Characteristics	Inter-G + nonhypo-A	Hypo-G	Inter-G	Hyper-G	Hypo-A	Hypo-G + Hypo-A	Inter-G + Hypo-A	Hyper-G + Hypo-A	SD
Cases: numbers	112	40	196	100	147	25	94	37	
Age	74.2 ± 15.3	75.9 ± 10.8	74.1 ± 15.2	72.8 ± 15.3	75.8 ± 12.5	75.1 ± 10.5	77.9 ± 12.0	73.2 ± 14.7	NS
Gender (male; %)	76/112 (67%)	21/40 (51%)	126/196 (65%)	57/100 (56%)	86/147 (59%)	13/25 (52%)	55/94 (59%)	22/37 (59%)	NS
APACHE II score	20.4 ± 6.7	28.7 ± 7.4**	21.5 ± 7.0	24.0 ± 6.5*	24.3 ± 7.7*	29.7 ± 7.8**	22.5 ± 7.1	24.0 ± 7.3	S
SOFA score	6.9 ± 2.8	10.0 ± 4.0**	7.1 ± 2.9	7.3 ± 2.6	8.0 ± 3.5	11.0 ± 4.3**	7.3 ± 3.0	7.1 ± 3.0	S
WBC (× 10^3^/μl)	11.2 ± 6.6	10.1 ± 7.8	11.9 ± 8.6	14.5 ± 7.5*	13.4 ± 10.1	10.5 ± 9.3	12.8 ± 10.5	16.5 ± 8.5*	S
Hemoglobin (g/dl)	12.4 ± 2.9	10.4 ± 2.5	12.0 ± 2.7	12.4 ± 2.5	11.2 ± 2.5	9.7 ± 2.3**	11.4 ± 2.4	11.5 ± 2.8	S
Hematocrit (%)	36.9 ± 8.3	31.3 ± 7.0*	35.8 ± 7.8	36.9 ± 7.2	33.6 ± 7.3*	29.1 ± 6.4**	34.2 ± 7.3*	34.7 ± 8.1	S
Platelet (× 10^3^/μl)	207.2 ± 11.5	168.0 ± 121.8	211.8 ± 119.1	238.6 ± 108.7	208.85 ± 129.1	140.1 ± 123.8*	215.2 ± 127.6	244.0 ± 130.0	S
Albumin (g/dl)	3.4 ± 0.5	2.5 ± 0.6**	2.9 ± 0.7**	2.9 ± 0.7**	2.2 ± 0.5**	2.1 ± 0.5**	2.3 ± 0.6**	2.2 ± 0.4**	S
T. bilirubin (mg/dl)	1.1 ± 1.4	1.7 ± 2.0	1.1 ± 1.4	0.8 ± 0.6	1.1 ± 1.5	1.8 ± 2.4	1.1 ± 1.3	0.7 ± 0.6	NS
GOT (U/l)	264.2 ± 1263.2	241.4 ± 482.3*	200.0 ± 964.7	85.4 ± 148.2	109.6 ± 169.5	143.0 ± 157.9	110.8 ± 190.9	74.6 ± 81.8	S
GPT (U/l)	108.0 ± 4966.8	102.8 ± 209.2	84.1 ± 379.3	53.3 ± 108.4	52.9 ± 78.3	53.3 ± 52.4	50.2 ± 75.1	53.4 ± 93.7	NS
Na (mEq/l)	139.7 ± 8.1	141.0 ± 9.9	140.1 ± 8.3	137.8 ± 9.0	139.8 ± 9.6	141.6 ± 11.7	140.7 ± 8.1	136.9 ± 10.4	NS
K (mEq/l)	4.4 ± 1.0	4.6 ± 1.3	4.4 ± 1.0	4.4 ± 1.0	4.4 ± 1.1	4.6 ± 1.4	4.4 ± 1.0	4.5 ± 1.1	NS
BUN (mg/dl)	42.0 ± 35.4	61.6 ± 42.4*	48.5 ± 38.3	47.8 ± 41.8	58.6 ± 43.2*	63.8 ± 39.4*	54.5 ± 39.4*	58.2 ± 51.8	S
Creatinine (mg/dl)	2.0 ± 2.1	2.8 ± 2.9	1.9 ± 1.9	1.9 ± 1.7	2.0 ± 1.8	2.7 ± 2.1	1.8 ± 1.5	2.0 ± 2.1	NS
Lactate (mmol/l)	3.9 ± 3.8	6.2 ± 8.7	4.7 ± 4.3	6.4 ± 4.1**	5.9 ± 5.7*	6.4 ± 10.2*	5.5 ± 4.6	6.0 ± 3.7	S
HCO^3−^ (mmol/l)	20.0 ± 5.7	20.3 ± 7.9	19.8 ± 5.5	18.8 ± 5.6	19.6 ± 5.6	20.5 ± 7.4	19.8 ± 5.0	19.0 ± 5.2	NS
CRP (mg/dl)	10.5 ± 10.0	11.1 ± 10.2	14.0 ± 11.6	13.0 ± 11.1	17.0 ± 11.5**	12.1 ± 9.8	18.0 ± 12.0*	15.8 ± 11.1	S
B.G (mg/dl)	149.9 ± 30.4	40.2 ± 20.2**	145.8 ± 32.1	324.6 ± 196.1**	189.2 ± 194.2	38.2 ± 19.9**	142.2 ± 33.9	400.7 ± 287.2**	S
Mortality (%)	17/112 (15.2%)	21/40 (52.5%)**	51/196 (26.0%)*	20/100(20.0%)	59/147 (40.2%)	15/25 (60.0%)**	35/94 (37.2%)*	10/37 (27.0%)	S

All groups, except for gender and mortality, were compared with the Inter-G + Nonhypo-A group (control) using Tukey–Kramer or Steel–Dwass’s post hoc test after one-way analysis of variance (ANOVA) or Kruskal–Wallis analysis. Significant difference between the group and Inter-G + Nonhypo-A are indicated by an asterisk

In the comparison of gender and mortality between the each group and Inter-G + Nonhypo-A, the Pearson exact test were performed due to categorical variables

*SD* statistical difference, *S* significant difference between groups compared to the control, *NS* no significant, *APACHE II* Acute Physiology and Chronic Health Evaluation II, *SOFA score* Sequential Organ Failure Assessment score, *T. bilirubin* total bilirubin, *WBC* white blood cell, *CRP* C-reactive protein, *Hypo-G* hypoglycemia group (blood glucose level < 80 mg/dl), *Hyper-G* hyperglycemia group (blood glucose level ≥ 200 mg/dl), *Inter-G* intermediate glycemia group (blood glucose level = 80–199 mg/dl), *Hypo-A* hypoalbuminemia (blood albumin < 2.8 mg/dl), *Nonhypo-A* non-hypoalbuminemia (blood albumin ≥ 2.8 mg/dl or more), *Inter-G + Nonhypo-A* patients with a glycemic level between 80 and 199 mg/dl and blood albumin 2.8 mg/dl or more, *Hypo-G + Hypo-A* patients with a glycemic level < 80 mg/dl and blood albumin < 2.8 mg/dl, *Inter-G + Hypo-A* patients with a glycemic level between 80 and 199 mg/dl and blood albumin < 2.8 mg/dl, *Hyper-G + Hypo-A* patients with a glycemic level ≥ 200 mg/dl and blood albumin < 2.8 mg/dl, In each Hypo-A, Inter-G + Nonhypo-A, Hypo-G + Hypo-A, Inter-G + Hypo-A and Hyper-G + Hypo-A group, there were some cases which were included in more than one group

*p < 0.05

**p < 0.0001

**Fig. 1:**
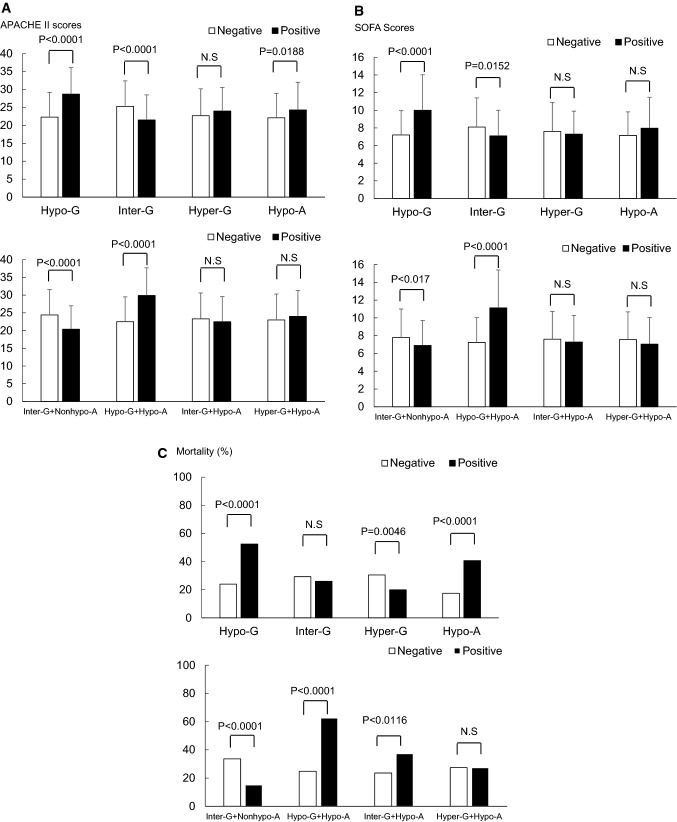
APACHE II scores, SOFA scores and mortality for each glycemic group and with or without hypoalbuminemia. **a** APACHE II scores. **b** SOFA scores. **c** Mortality. Significant differences in APACHE II scores, SOFA scores and mortality are observed between In the Hypo-G and Hypo-G + Hypo-A groups and the other groups in cases with hypoalbuminemia (Positive: black bars), compared to cases without hypoalbuminemia (No: white bars in figure). APACHE II score and SOFA score were calculated using initial data from the emergency department. *APACHE II score* Acute Physiology and Chronic Health Evaluation score II, *SOFA score* sequential Organ Failure Assessment score, *Negative* patients who did not meet the classification criteria for the specified group are indicated by white bars, *Positive* patients who met the classification criteria for the specified group are indicated by black bars, *Hypo-G* hypoglycemia group (blood glucose level < 80 mg/dl), *Hyper-G* hyperglycemia group (blood glucose level ≥ 200 mg/dl), *Inter-G* intermediate glycemia group (blood glucose level = 80–199 mg/dl), *Hypo-A* hypoalbuminemia (blood albumin < 2.8 mg/dl), *Nonhypo-A* non-hypoalbuminemia (blood albumin ≥ 2.8 mg/dl or more), *Inter-G + Nonhypo-A* patients with a glycemic level between 80 and 199 mg/dl and blood albumin 2.8 mg/dl or more, *Hypo-G + Hypo-A* patients with a glycemic level < 80 mg/dl and blood albumin < 2.8 mg/dl, *Inter-G + Hypo-A* patients with a glycemic level between 80 and 199 mg/dl and blood albumin < 2.8 mg/dl, *Hyper-G + Hypo-A* patients with a glycemic level ≥ 200 mg/dl and blood albumin < 2.8 mg/dl. Some cases in each Inter-G + Norhypo-A, Hypo-G + Hypo-A, Inter-G + Hypo-A and Hyper-G + Hypo-A group were included in more than one group

Figure 1 shows APACHE II scores (Fig. 1a), SOFA scores (Fig. 1b), and mortality (Fig. 1c) among each glycemic group and patients with or and without hypoalbuminemia. In particular, significantly higher scores and mortality are observed in the Hypo-G (*p* < 0.0001) and Hypo-G + Hypo-A (*p* < 0.0001) groups when classification criteria were met (black bars), compared to cases whose classification was not met (white bars).

APACHE II or SOFA scores were used to determine whether the Hypo-G + Hypo-A group was associated with mortality, according to ROC curve-derived cut-off values. According to the ROC curve of the APACHE II or SOFA scores in the Hypo-G + Hypo-A group, AUC is 0.76 for the APACHE II score (sensitivity 0.75, 1-specificity 0.42), for the APACHE II score value of 26, and AUC is 0.78 for the SOFA score (sensitivity 0.71, 1-specificity 0.42) for the SOFA score value of 9.

Finally, a single logistic regression analysis for the initial laboratory data at emergency department admission shows that APACHE II score (OR 1.116; 95% CI 1.0753–1.1611, *p* < 0.0001), SOFA score (OR 1.256; 95% CI 1.1559–1.3737, *p* < 0.0001), Hypo-G (OR 3.503; 95% CI 1.7820–6.9351, *p* = 0.0003), Hypo-A (OR 3.243; 95% CI 1.9774–5.3957, *p* < 0.0001) or Hypo-G + Hypo-A (OR 5.065; 95% CI 2.1664–12.498, *p* = 0.0002), existing in each septic patient, is associated with higher mortality (Table 5).

**Table 5:** ICU mortality and each glycemic condition with/without hypoalbuminemia

Glycemic conditions	Odds	95% CI	*p* value
Hypo-G	3.503	1.7820–6.9351	0.0003
Inter-G	1.175	0.7324–1.9093	0.5083
Hyper-G	1.756	1.0150–3.1443	0.0439
Hypo-G + Hypo-A	5.065	2.1664–12.498	0.0002
Inter-G + Hypo-A	1.925	1.1487–3.2115	0.0131
Hyper-G + Hypo-A	0.980	0.4349–2.0552	0.9591
^a^Inter-G + Nonhypo-A	0.349	0.1897–0.6152	0.0002
Other parameters			
APACHE II score	1.116	1.0753–1.1611	< 0.0001
SOFA score	1.256	1.1559–1.3737	< 0.0001
Hypo-A	3.243	1.9774–5.3957	< 0.0001

Odds ratio of each glycemic condition, SOFA score, and/or hypoalbuminemia, respectively, were independently calculated by a single logistic regression analysis of the initial laboratory data at the time of admission due to multicollinearity. In each Hypo-A, Inter-G + Nonhypo-A, Hypo-G + Hypo-A, Inter-G + Hypo-A and Hyper-G + Hypo-A group, there were some cases which were included in more than one group

*Hypo-G* hypoglycemia group (blood glucose level < 80 mg/dl), *Hyper-G* hyperglycemia group (blood glucose level ≥ 200 mg/dl), *Inter-G* intermediate glycemia group (blood glucose level = 80–199 mg/dl), *Hypo-A* hypoalbuminemia (blood albumin < 2.8 mg/dl), *Nonhypo-A* non-hypoalbuminemia (blood albumin ≥ 2.8 mg/dl or more), *Inter-G + Nonhypo-A* patients with a glycemic level between 80 and 199 mg/dl and blood albumin 2.8 mg/dl or more, *Hypo-G + Hypo-A* patients with a glycemic level < 80 mg/dl and blood albumin < 2.8 mg/dl, *Inter-G + Hypo-A* patients with a glycemic level between 80 and 199 mg/dl and blood albumin < 2.8 mg/dl, *Hyper-G + Hypo-A* patients with a glycemic level ≥ 200 mg/dl and blood albumin < 2.8 mg/dl, *SOFA* sequential organ failure assessment

Odds ratio was higher in the “Hypo-G + Hypo-A” group than any other glycemic condition

^a^Inter-G + Nonhypo-A: Patients classified in this group showed a lower odds ratio for mortality

## Discussion

Due to high morbidity or mortality during intensive care, prompt clinical evaluation of severity in sepsis is an important strategy to improve outcome [17, 18]. To evaluate severity and mortality in critical illness, APACHE II score and SOFA score are generally used in the ICU. This study indicates a higher OR for mortality risk in patients with hypoglycemia (OR 3.503), hypoalbuminemia (OR 3.243), or both hypoglycemia and hypoalbuminemia (OR 5.065), than that estimated by SOFA score (OR 1.256). These results may be useful for early and easy identification of high-mortality risk in patients with sepsis at the time of hospital admission.

During systemic excess stress insults, blood glucose level usually increases by insulin resistance or secretion of adrenal hormones such as catecholamine [19–21]. These increased levels of glucose, or stress-induced hyperglycemia, is a normal and important physiologic response to stress that is common in critically ill patients [22, 23]. Hypoglycemia, however, has been considered a critical pathophysiological condition in pneumonia [3, 4] and in critically ill patients [24, 25]. There are reports that hypoglycemia is also an available prognosticator in patients with sepsis [26–28]. Previous reports, together with our results, demonstrate the hypoglycemia observed in the initial data at the hospital admission may be useful in indicating severity and in the prognostic prediction in septic patients. Hypoalbuminemia is in itself an effective and powerful indicator of mortality and morbidity in cases of sepsis [5]. Moreover, hypoglycemia under hypoalbuminemia conditions in septic patients should be considered as an alert sign for clinicians to provide early management.

The pathophysiology of hypoglycemia or hypoalbuminemia may have many mechanisms. In the experimental model of sepsis, glucose utilization would increase in macrophage-rich tissues, such as the liver, and then lead to hypoglycemia [29, 30] and decreased hepatic glucose production [30, 31]. Reduction in albumin synthesis by inflammatory reactions, such as many mediators, is well known [32, 33]. Vascular permeability increases during sepsis, leading to the transcapillary loss of albumin and acceleration to hypoalbuminemia [33–35]. Taken together, these patients may experience depression with hepatic gluconeogenesis, as a result of decreased sensitivity to stress hormones or adrenal failure [30] leading to hypoglycemia, and concomitant with reduced albumin synthesis and transcapillary loss caused by inflammatory reactions, depending on the severity of inflammation.

Although age or cardiac function is also an incremental prognostic value [36, 37], for early evaluation of sepsis, quick SOFA score and screening for patients suspected of having sepsis can be expected to start treatment earlier [38]. If the severity of sepsis and outcomes could be predicted using the initial simple data at admission after evaluation of sepsis using quick SOFA, physicians could provide a therapeutic plan earlier, in combination with various additional treatments for patients in the high-mortality group.

There are some limitations to this study. A major limitation is that this is a retrospective observational study of a limited number of patients in a single institution, so detailed information of patient nutritional conditions, such as body mass index and duration of illness before hospitalization, was not evaluated. This study also did not consider history of diabetes or the effect of diabetic agents since some patients had not received a medical check for a long time, and other patients had been diagnosed diabetes but did not take their medication. In the emergency department, glycemic value evaluation is greatly influenced by nutritional intake and antidiabetic therapy. Rather than acute onset, septic conditions continued for days and gradually deteriorated. Hence, accurate data regarding nutritional intake or antidiabetic medicine in cases of patients with diabetic history could not be obtained. Generally, estimating illness duration is difficult since many septic patients do not know the time of onset. Due to these reasons, this study could only identify patients who had a long duration of illness as reflected in collapsed pathophysiological conditions after systemic depletion due to infection. In addition, although hyperglycemia in septic patients at admission is well known to carry a high mortality risk, diabetes is not [22, 39, 40]. In this study, bias in initial blood glucose or albumin levels was present. Actually, data were obtained from patients only at the time of admission. The underlying mechanisms regarding how hypoglycemia in septic patients under hypoalbuminemia conditions is related to outcome still remain unclear. Finally some cases are included in more than one group. For multicollinearity reasons, multiple regression analysis using a multivariate model could not be performed for outcome evaluation. The odds ratio of mortality was calculated by single logistic regression analysis of each group.

## Conclusion

Hypoglycemia with hypoalbuminemia at admission is related to higher ICU mortality in septic patients, compared with other glycemic conditions. A combination of simple laboratory data from the emergency may be useful to predict the severity and mortality after diagnosis of sepsis. Physicians should be keenly aware of these conditions to provide immediate intensive treatment after diagnosis of sepsis. Hypoglycemia with hypoalbuminemia may contribute to the underlying mechanisms in septic patients.
